# KR-12 Derivatives Endow Nanocellulose with Antibacterial and Anti-Inflammatory Properties: Role of Conjugation Chemistry

**DOI:** 10.1021/acsami.3c04237

**Published:** 2023-05-11

**Authors:** Anna Blasi-Romero, Molly Ångström, Antonio Franconetti, Taj Muhammad, Jesús Jiménez-Barbero, Ulf Göransson, Carlos Palo-Nieto, Natalia Ferraz

**Affiliations:** † Division of Nanotechnology and Functional Materials, Department of Materials Science and Engineering, Uppsala University, P.O. Box 35, SE-75103 Uppsala, Sweden; ‡ CIC bioGUNE, Derio-Bizkaia 48160, Spain; § Pharmacognosy, Department of Pharmaceutical Biosciences, Biomedical Centre, Uppsala University, P.O. Box 591, SE-75124 Uppsala, Sweden; ∥ Department of Inorganic & Organic Chemistry, Faculty of Science and Technology, University of the Basque Country, Leioa 48940, Spain; ⊥ IKERBASQUE, Basque Foundation for Science and Technology, Bilbao 48009, Spain; # Centro de Investigacion Biomedica En Red de Enfermedades Respiratorias, Madrid 28029, Spain

**Keywords:** cellulose nanofibrils, antimicrobial peptides, chronic wounds, wound healing, peptide immobilization, molecular dynamics simulations

## Abstract

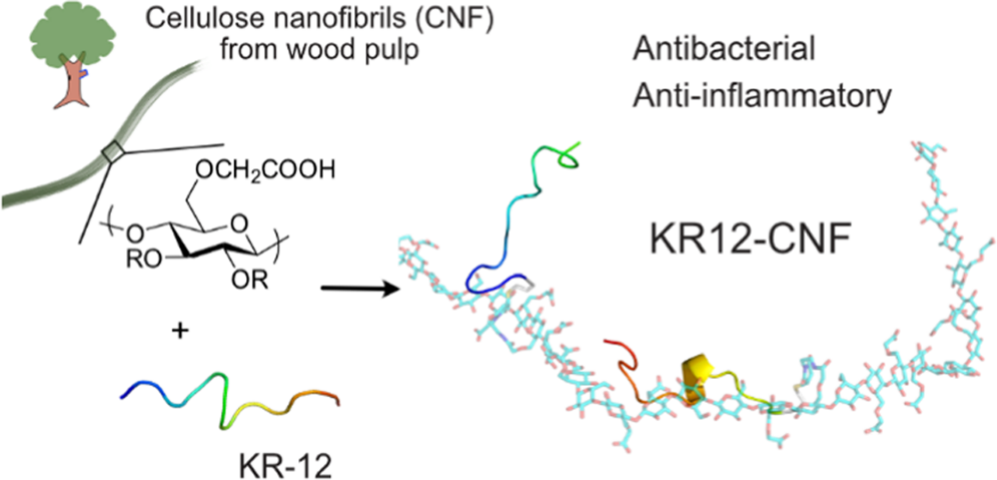

This work combines the wound-healing-related properties of the host defense peptide KR-12 with wood-derived cellulose nanofibrils (CNFs) to obtain bioactive materials, foreseen as a promising solution to treat chronic wounds. Amine coupling through carbodiimide chemistry, thiol-ene click chemistry, and Cu(I)-catalyzed azide-alkyne cycloaddition were investigated as methods to covalently immobilize KR-12 derivatives onto CNFs. The effects of different coupling chemistries on the bioactivity of the KR12-CNF conjugates were evaluated by assessing their antibacterial activities against *Escherichia coli* and *Staphylococcus aureus*. Potential cytotoxic effects and the capacity of the materials to modulate the inflammatory response of lipopolysaccharide (LPS)-stimulated RAW 245.6 macrophages were also investigated. The results show that KR-12 endowed CNFs with antibacterial activity against *E. coli* and exhibited anti-inflammatory properties and those conjugated by thiol-ene chemistry were the most bioactive. This finding is attributed to a favorable peptide conformation and accessibility (as shown by molecular dynamics simulations), driven by the selective chemistry and length of the linker in the conjugate. The results represent an advancement in the development of CNF-based materials for chronic wound care. This study provides new insights into the effect of the conjugation chemistry on the bioactivity of immobilized host defense peptides, which we believe to be of great value for the use of host defense peptides as therapeutic agents.

## 1 Introduction

Chronic wounds are a burden for healthcare systems and significantly affect the quality of patients’ lives. These wounds are characterized by elevated levels of reactive oxygen species (ROS), proinflammatory cytokines, and degradative proteases in the wound environment. Unresolved inflammation, impaired fibroblast function, decreased angiogenesis, and harmed extracellular matrix deposition are other hallmarks of nonhealing wounds.^1^ Opportunistic pathogens colonize chronic wounds, forming biofilms and further contributing to the imbalanced environment.^2,3^ Toxins released from the bacteria trigger the recruitment of immune cells, further exacerbating the inflammatory response.^4^ Chronic wound care demands treatments able to correct the imbalances in the wound; examples include targeting the immune response by promoting macrophage polarization and inhibiting proinflammatory cytokines, reducing the excess of ROS and proteases and fighting bacterial infections.^5,6^

Host defense peptides (HDPs) are naturally occurring polypeptides with sequences of 12–50 residues that mediate a wide range of biological processes.^7^ Several studies have demonstrated the role of HDPs in wound healing via multiple actions, including the modulation of cytokine production and the promotion of skin cell migration and proliferation.^4,8^ The ability of HDPs to fight bacterial infections and biofilm formation makes them ideally suited to treat chronic wounds, as substitutes to conventional antibiotics.^4^ This dual action of HDPs, in promoting wound healing while also targeting bacterial growth, makes them promising therapeutic agents to treat chronic wounds.

The human HDP cathelicidin LL-37, expressed in neutrophils and keratinocytes among other cells, has gained attention because of its well-documented role in accelerating wound healing.^9−11^ However, the harsh chronic wound environment represents a challenge for the use of LL-37 and other HDPs since these peptides are highly sensitive to proteolytic degradation. Although HDPs are generally considered safe, their interaction with eukaryotic cell membranes can lead to cytotoxicity. This risk of cytotoxicity increases when high doses of HDP are used to compensate for the peptides’ short half-life.^12^ To address these limitations, delivery systems that protect the peptide from the proteolytic wound environment and allow the targeted and sustained release of the HDP are being developed.^13,14^ Another possible route being explored is the immobilization of the peptide onto the surface of biomedical materials.^15−17^ One more limitation of using LL-37 as a therapeutic agent is the high manufacturing cost associated with the production of long peptides.^12,18^ To overcome this drawback, truncated LL-37 fragments are being investigated.^18−21^ The residual peptide KR-12 was found to be the shortest fragment of LL-37 to retain its antimicrobial function.^19^ KR-12 is also known to modulate inflammation by binding and neutralizing lipopolysaccharides (LPS).^22,23^

Wood-derived cellulose nanofibrils (CNFs) are a promising material for the development of wound dressings.^24,25^ This nanomaterial consists of individual fibrils with a diameter of 2–10 nm and a length of several micrometers that arrange into 20–50 nm thick aggregates. CNFs form colloidal suspensions in water with a three-dimensional (3D) network maintained by the entanglement of the fibers, hydrogen bonds, and van der Waals and electrostatic interactions.^26^ CNFs are renewable materials derived from nonanimal origins with tunable aspect ratio, surface charge, and physical forms (CNFs can be prepared as gel suspensions, self-standing hydrogels, foams, or films). These properties have led to CNFs being a topic of intense interest in wound care.^24,25,27^ The in vitro and in vivo wound-healing properties of CNF-based materials have been demonstrated in acute wound models and burns treatment.^28−30^ In these works, CNF-based wound dressings have improved the time of reepithelialization, were ease to apply, and provided a desirable moist healing environment. However, at the time of writing, the potential of CNFs to accelerate the healing of chronic wounds is not proven.

Ongoing research is focused on expanding the use of CNF-based materials in chronic wound care. The wide array of possible chemical modifications on CNFs can be exploited to functionalize the nanofibers with bioactive molecules, the aim of which is to endow the nanofibers with the ability to modify the chronic wound environment and actively promote wound healing.

In this work, we functionalize CNFs with KR-12 as a strategy to develop nanocellulose-based chronic wound care dressings. Moreover, the immobilization of the peptide on the nanofibers is expected to increase the peptide’s stability toward proteases, which in turn is anticipated to increase the therapeutic potential of HDPs.^15,16^

The covalent immobilization of KR-12 onto CNFs via three different chemical approaches has been explored. KR-12 derivatives were designed to evaluate the effects of different coupling chemistries on the activity of the resulting KR12-CNF materials. The effect of amino acid substitutions on the activity of the immobilized peptide was also investigated. The degree of peptide substitution, antibacterial properties, cytotoxicity, and immunomodulatory capacity of the KR12-CNF conjugates were all analyzed and reported. Molecular dynamics (MD) simulations were performed to obtain an insight into the peptide exposure and conformation when immobilized onto CNFs.

## 2 Materials and Methods

### 2.1 Materials

#### 2.1.1 Chemicals and Reagents

*N*-(3-Dimethylaminopropyl)-*N′*-ethylcarbodiimide hydrochloride (EDC), *N*-hydroxysuccinimide (NHS), *N*-(2-aminoethyl) maleimide (AEM), propargyl bromide (80% in toluene), CuSO_4_·5H_2_O, and ascorbic acid were purchased from Sigma-Aldrich Sweden AB, Sweden, and used as received. Fetal bovine serum (FBS), penicillin, streptomycin, presto blue cell viability reagent, Dulbecco’s phosphate buffered saline (PBS), Dulbecco’s modified eagle medium (DMEM) with high glucose, TNF-α mouse High Sensitivity ELISA Kit (BMS607HS), and Pierce BCA Protein Assay Kit were bought from Thermo Fisher Scientific (Waltham, MA). Dimethyl sulfoxide (DMSO, ≥99.5%), lipopolysaccharides (LPS) O111:B4 from *Escherichia coli*, Luria-Bertani (LB) agar (Miller), Tryptic Soy Broth (TSB), CelLytic M, and Tris(hydroxymethyl)-aminomethane (Tris·HCl) were obtained from Sigma-Aldrich (St. Louis, MO).

#### 2.1.2 Peptide Design and Synthesis

KR-12 derivatives were designed with their method of conjugation to CNF in mind. The peptides amKR-12, amD9AKR-12, cysKR-12, and N_3_KR-12 were assembled using fluorenylmethyloxycarbonyl (Fmoc)-based solid-phase peptide synthesis with piperidine (20% v/v in dimethylformamide) as the Fmoc deprotecting agent, on a CEM Liberty 1 automated microwave-assisted peptide synthesizer, using methods previously described elsewhere.^21,31^ All peptides were amidated at the terminal carboxylic acid in order to increase the positive net charge of the peptide and decrease the risk of side reactions. Peptides were purified by preparative reverse-phase high-pressure liquid chromatography (RP-HPLC). Peptide purity was >95% as judged by analytical RP-HPLC-UV (215 nm), and their identity was confirmed using mass spectrometry (MS). Peptide sequences are provided in Table 1 and their structures are illustrated in Figure S1. Analytical HPLC traces and MS spectra are presented in Figure S2.

**Table 1:** Peptide Sequences and KR12-CNF Materials Investigated

peptide sequence	peptide name	synthetic route^a^	corresponding material	peptide content^b^
KRIVQRIKDFLR-NH_2_	amKR-12	a	amKR12-CNF	0.18 ± 0.02
KRIVQRIKAFLR-NH_2_	amD9AKR-12	a	amD9AKR12-CNF	0.19 ± 0.04
CPGG-KRIVQRIKDFLR-NH_2_	cysKR-12	b	cysKR12-CNF	0.17 ± 0.03^c^
N_3_-KRIVQRIKDFLR-NH_2_	N_3_KR-12	c	N_3_KR12-CNF	0.16 ± 0.01

a Depicted in Figure 1. Synthetic route “a” corresponds to amine coupling by EDC/NHS activation, “b” to amine coupling followed by thiol-ene reaction, and “c” to Cu(I)-catalyzed azide-alkyne cycloaddition.

b mmol peptide/g CNF, determined by elemental analysis of the nitrogen content.

c Unreacted intermediates (maleimide-CNF or its derivatives) are not expected to significantly affect the peptide quantification by the total nitrogen content.

#### 2.1.3 Nanocellulose Materials

CNFs (RISE Bioeconomy, Stockholm, Sweden) were produced from commercial, never-dried, bleached, sulfite softwood dissolving pulp (lignin content < 1.5%, xylose < 1.7%, mannose < 1.8%, Domsjö Fabriker AB, Stockholm, Sweden). Unmodified-CNF (u-CNF) was prepared by enzymatic pretreatment of the wood pulp^32^ and carboxymethylated CNF (c-CNF, 1800 μmol carboxyl groups per gram of dry CNF) was prepared by the method described by Hua et al.^33^ The CNF materials were pretreated using the method described by Nordli et al.^34^ before functionalization with peptides to lower the endotoxin load. Briefly, the CNF materials were resuspended in 100 mM NaOH and autoclaved for 2 h. Thereafter, the suspension was washed three times with sterile MilliQ water. After the pretreatment, an endotoxin level of 0.54 ± 0.04 EU/mg CNF was quantified with a LAL (limulus amebocyte lysate) assay.

### 2.2 Functionalization of CNFs with KR-12 Derivatives

KR-12 derivatives were covalently incorporated onto CNFs via three different chemical approaches.

#### 2.2.1 Amine Coupling through EDC/NHS Activation

The peptides amKR-12 and amD9AKR-12 were incorporated onto c-CNF via EDC/NHS coupling chemistry (Figure 1a). A suspension of c-CNF (7.5 g of 2 wt % suspension, 0.150 g of dry c-NFC, 0.27 mmol COOH) was homogenized by stirring in deionized water. Next, 3 mL of an aqueous solution containing EDC (103.5 mg, 0.54 mmol) and NHS (93.2 mg, 0.81 mmol) was added to the suspension, and the pH was adjusted to 5.5–6.0 by adding 0.1 M HCl and left stirring for 30 min to activate the carboxylic acids. Afterward, the suspension was centrifuged in order to remove the residual EDC and NHS. The activated c-CNF was resuspended in deionized water and a solution containing the peptide (848.3 mg of amKR-12 or 824.6 mg of amD9AKR-12, 0.54 mmol) was added dropwise to the suspension. The pH was adjusted to 7.5–8.0 by adding 0.1 M NaOH and the reaction mixture was allowed to proceed overnight at 25 °C with continuous stirring. To purify, the modified material was washed three times with 0.01 M NaOH (pH = 12) and three times with water in order to remove the unreacted peptide that could potentially be entrapped within the nanofibers, as well as any impurities.^35^

**Figure 1:**
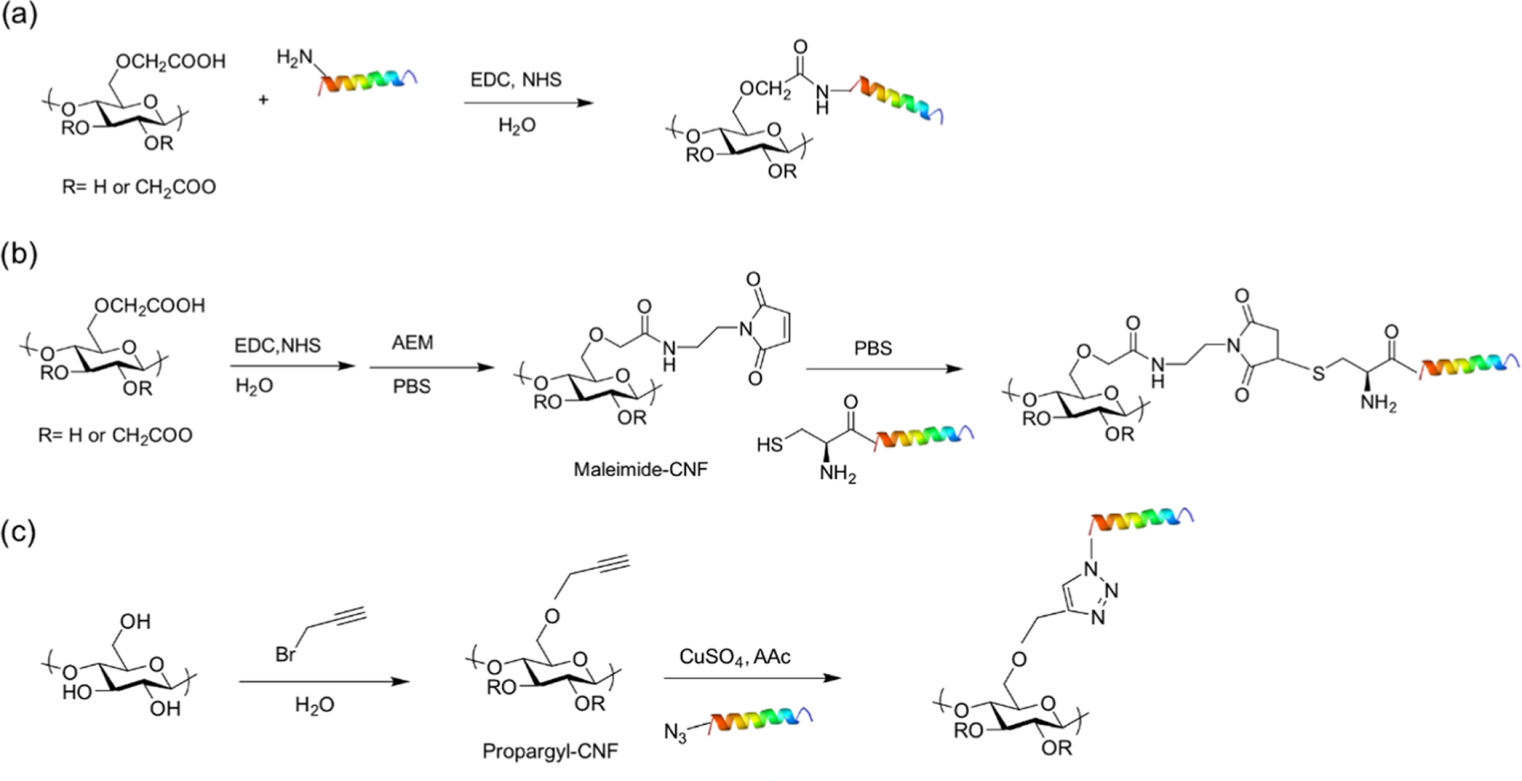
Functionalization of CNFs with KR-12 derivatives via three synthetic routes: (a) amine coupling through EDC/NHS activation, (b) amine coupling followed by the thiol-ene reaction, and (c) Cu(I)-catalyzed azide-alkyne cycloaddition.

#### 2.2.2 Amine Coupling Followed by Thiol-Ene Click Chemistry

cysKR-12 was conjugated to c-CNF via a thiol-ene click reaction (Figure 1b). For this, a suspension of c-CNF (7.5 g of 2 wt % suspension, 150 mg of dry c-NFC, 0.27 mmol COOH) was homogenized by stirring in deionized water. Next, 3 mL of an aqueous solution containing EDC (103.5 mg, 0.54 mmol) and NHS (93.2 mg, 0.81 mmol) was added to the suspension and the pH was adjusted to 5.5–6.0 with 0.1 M HCl before stirring for 30 min to activate the carboxylic acids. Afterward, the suspension was centrifuged in order to remove the residual EDC and NHS. The activated c-CNF was resuspended in PBS (pH = 7.4) and then reacted with *N*-(2-aminoethyl) maleimide (AEM, 137.2 mg, 0.54 mmol) at 25 °C for 2 h, followed by three washes with deionized water. Next, the maleimide-CNF conjugate (150 mg, 0.10 mmol maleimide, 0.69 mmol maleimide/g material) was resuspended in PBS and reacted with a solution of cysKR-12 (188.5 mg, 0.10 mmol) in PBS for 4 h at 25 °C. Lastly, the modified CNF was washed three times with 0.1 M NaOH/0.05 M NaHCO_3_ buffer (pH = 11), and three times with deionized water in order to remove the unreacted peptide potentially entrapped within the nanofibers, as well as impurities.

#### 2.2.3 Cu(I)-Catalyzed Azide-Alkyne Cycloaddition

N_3_KR-12 was conjugated to u-CNF via Cu(I)-catalyzed azide-alkyne cycloaddition (CuAAC) (Figure 1c). A suspension of u-CNF (40 g of 2 wt % suspension, 0.80 g of dry u-CNF, 5 mmol anhydroglucose units, AGUs) was prepared in 80 mL of 0.28 M NaOH and stirred for 1 h at 25 °C. Then, propargyl bromide (2.67 mL, 25 mmol, 5:1 molar equiv to the AGUs) was added to the NaOH-activated CNF and the reaction mixture was stirred overnight at 25 °C. For purification, the modified material was washed three times with EtOH and three times with deionized water. In a second step, the previously prepared propargyl-CNF (30 g of 0.5 wt %, 0.150 g of dry content) was dispersed in water to which a solution of N_3_KR-12 (319.2 mg, 0.2 mmol) was added. Next, a freshly prepared solution of CuSO_4_·5H_2_O (56.2 mg, 0.22 mmol) and ascorbic acid (AAc, 78.5 mg, 0.44 mmol) in 3 mL of deionized water was added and the resulting reaction mixture was stirred overnight in dark at 25 °C. To purify, the final material was washed three times with EtOH and three times with deionized water to remove the unreacted reagents.^36^

### 2.3 Degree of Substitution

The peptide content of the KR12-CNF materials was determined by elemental analysis of the total nitrogen content. For this, dry KR12-CNF samples were submitted to MEDAC Ltd., analytical and chemical consultancy services (Cobham, U.K.), where a Thermo FlashEA 1112 instrument was used for nitrogen quantification. The weight percentage of nitrogen in KR12-CNF was converted to mmol peptide/g CNF.^37,38^

### 2.4 Antibacterial Properties of KR12-CNF Materials

Two bacterial strains, one Gram-negative, *E. coli* (ATCC 25922), and one Gram-positive, *S. aureus* (ATCC 29213), were selected to evaluate the antibacterial properties of the KR12-CNF materials. Bacterial cultures were started from frozen stocks and left to grow overnight in 3% TSB at 37 °C until they reached their mid-logarithmic phase. The bacteria were then washed and resuspended in Tris·HCl buffer (10 mM Tris base adjusted with HCl to pH 7.4 at 37 °C).

#### 2.4.1 Measurement of Bacterial Growth in the Presence of KR12-CNF Materials

KR12-CNF suspensions from 50 to 1000 μg/mL were prepared in Tris·HCl buffer on the day of the experiment. Then, 50 μL of the KR12-CNF suspension was inoculated with 50 μL of the bacterial suspension (in Tris·HCl) in a 96-well transparent plate to obtain an optical density at 600 nm (OD600) of 0.1 (which corresponded to 2 × 10^13^ and 1.6 × 10^11^ CFU/mL for *E. coli* and *S. aureus*). These mixtures were incubated for 1 h at 37 °C and are henceforth referred to as bacterial exposure to the materials at low salt concentration (LSC). Next, 5 μL of 20% TSB was added to each well and incubated at 37 °C (this was considered to be the time point 0 of the exposure in TSB conditions). Bacterial growth was monitored by measuring the OD600 every hour for 6 h with a plate reader (Varioskan Flash plate reader, ThermoScientific (Waltham, MA)). Bacterial growth during the exponential growth phase (3 and 5 h for *E. coli* and *S. aureus*, respectively) was expressed as a percentage of the negative control.

The free peptides were evaluated in parallel, at concentrations equivalent to the peptide content in the KR12-CNF suspensions, from 10 to 200 μM. c-CNF was used as a reference and bacteria cultured in the absence of any CNF material were used a negative control.

#### 2.4.2 Colony Forming Unit (CFU) Assay

The number of viable cells after incubation with the different KR12-CNF conjugates was assessed by a colony count assay. Bacteria were exposed to the different KR12-CNF materials (500 and 1000 μg/mL) as described above, and agar plates were then streaked with these bacteria after 1 h of incubation in LSC, and in the exponential growth phase (2.5 h for *E. coli* and 4 h *S. aureus*) when cultured in TSB. For this, 10 μL was aliquoted from each well, then serially diluted with Tris·HCl before streaking on agar plates. Plates were left at 37 °C overnight and the CFUs were counted manually. Results are given as the mean ± standard error of the mean from at least three independent experiments with samples run in triplicate.

### 2.5 Macrophage Interactions with KR12-CNF Materials

Mouse macrophages, RAW 264.7 (ATCC TIB-71), from American Type Culture Collection (ATCC, Manassas, VA), were cultured in DMEM supplemented with 10% v/v FBS, 100 U/mL penicillin, and 100 μg/mL streptomycin. Cells were cultured at 37 °C and 5% CO_2_ in a humidified atmosphere and passaged at 80% confluency by cell scraping.

#### 2.5.1 Cytotoxicity Study

The cytotoxicity of the KR12-CNF conjugates was assessed by exposing RAW 264.7 cell monolayers to suspensions of the materials. Cells were seeded at a density of 2 × 10^4^ cells/well in a 96-well plate and cultured for 24 ± 2 h in a 37 °C and 5% CO_2_ incubator with a humidified atmosphere. Thereafter, cell monolayers were exposed to 200 μL of KR12-CNF suspensions, at concentrations ranging from 250 to 1000 μg/mL, prepared in DMEM from 7 mg/mL stock suspensions.

The free peptides were assayed in parallel at concentrations equivalent to the peptide content in the respective KR12-CNF suspensions, from 50 to 200 μM. Unexposed cells were used as negative controls, cells exposed to 2.5% w/w DMSO in the cell culture medium were used as a positive control, and c-CNF was used as a reference in these cytotoxicity evaluations. The cytotoxicities of the intermediate species cysKR12-CNF and N_3_KR12-CNF (maleimide-CNF and propargyl-CNF, respectively; see Figure 1) were evaluated under the same conditions as the KR12-CNF suspensions.

Cell metabolic activity, total protein content, and cell membrane integrity after 24 ± 2 h of exposure were evaluated as indicators of cell cytotoxicity. The presto blue assay was used to evaluate the cell metabolic activity. The cell culture supernatant was replaced by 200 μL of 10% (v/v) presto blue in DMEM, and incubated for 90 min at 37 °C. Then, 100 μL aliquots were transferred to a black 96-well plate and the fluorescence measured at 560/590 nm excitation/emission in a microplate reader (Infinite M200 TECAN, Zurich, Switzerland). Cell metabolic activity was expressed as a percentage of the negative control.

The total protein in cell lysates was quantified using a BCA protein kit. After the cell culture supernatant was collected, the cell monolayers were carefully washed three times with warm PBS and then 200 μL of CelLytic M reagent was added to each well, followed by one freeze–thaw cycle. Lysates were centrifuged at 14,000 rcf for 15 min to remove cell debris, and the supernatants were collected for protein content evaluation. For this, 25 μL of each sample was added to 200 μL of BCA reagent in a 96-well plate, and the plate was shaken at 250 rpm for 30 s, followed by 1 h of incubation at 37 °C. The absorbance at 562 nm was measured in a microplate reader (Infinite M200 TECAN, Zurich, Switzerland). A calibration curve was prepared with bovine serum albumin (BSA) diluted in CelLytic M reagent and the protein concentration was extrapolated from this calibration curve.

The cell membrane integrity was evaluated by calcein-AM/propidium iodide staining followed by fluorescence microscopy imaging. A live/dead cell double staining kit suitable for fluorescence was used according to the manufacturer’s protocol. In brief, the cell supernatant was aspirated, 200 μL of staining solution (1:500 calcein-AM and 1:1000 propidium iodide in PBS) was added to each well, and the plate was incubated for 10 min at 37 °C. After incubation, the staining solution was removed and 200 μL of DMEM was added to each well. The samples were then imaged with a fluorescence microscope (Eclipse Ti-U Nikon, Tokyo, Japan).

#### 2.5.2 Modulation of LPS-Induced Inflammation

The capacity of the KR12-CNF conjugates to modulate LPS-induced inflammation in RAW 264.7 cells was assessed by treating LPS-stimulated cell monolayers with suspensions of KR12-CNF. For this, KR12-CNF suspensions were prepared as described in Section 2.5.1, and LPS was added at a final concentration of 10 ng/mL. Cell cytotoxic response was evaluated 24 ± 2 h after exposure. For samples that did not show a cytotoxic response, TNF-α content was quantified as a measurement of the inflammatory response. To do this, cell culture supernatants were collected by centrifuging at 14,000 rcf for 10 min to remove the cell debris and the levels of TNF-α were subsequently quantified using a TNF-α mouse ELISA kit following the protocol provided by the manufacturer. The TNF-α concentration was normalized against the total protein content.

### 2.6 Statistical Analysis

Results are expressed as the mean ± standard error of the mean from at least three independent experiments with samples run in triplicate. GraphPad Prism was used for the statistical analysis, and significant differences were considered for a confidence interval of 95%.

Assumptions for parametric tests were checked and log transformations were used to normalize data when required. A one-way ANOVA with Dunn’s test for multiple comparisons was used for parametric data. The Kruskal–Wallis test and Wilcoxon signed-rank test were used for nonparametric data.

### 2.7 Molecular Dynamics Simulations

MD simulations were performed to evaluate the accessibility and conformation of the KR-12 derivatives when immobilized onto CNFs. Furthermore, MD simulations were also used to obtain information about the reactivity of different lysine amino acids when peptide conjugation takes place by amine coupling via EDC/NHS activation. Details of the models, simulations, and data analysis are presented in the Supporting Information.

## 3 Results and Discussion

### 3.1 Material Synthesis and Characterization

The combination of HDPs with biomaterials has been suggested as a strategy to tailor the material activity, such as modulating the immune response or endowing antibacterial properties to the material.^17^ The interaction of the peptide with the material can take place via passive adsorption, entrapment, or covalent immobilization. The latter minimizes peptide leaching, can increase the long-term stability of the peptides, and may decrease their toxicity to mammalian cells. However, covalent immobilization can be detrimental to the activity of the HDP by affecting peptide conformation and reducing its availability to interact with bacterial membranes.^15,17^ Parameters such as the degree of peptide substitution, peptide orientation, conformation, use of spacers to connect the peptide with the material and the nature of the surface matrix will affect the bioactivity of HDP-biomaterial conjugates.^15,17^ Therefore, the surface chemistry used for the covalent immobilization can determine the properties of the HDP-material conjugate.

To develop bioactive CNF-based wound dressings, three different chemical approaches to covalently immobilize the HDP KR-12 onto the nanofibers were investigated in this work. The aim of exploring different synthetic routes was to attempt to control the activity of the conjugate by investigating selective and nonselective conjugation approaches. Moreover, efforts were made to keep the overall charge of the peptide positive, which is generally considered important for the antibacterial activity of HDPs.^7,39^

In the first synthetic route, amKR-12 and amD9AKR-12 were immobilized onto c-CNF via amidation of the primary amines in the Lys amino acids. These two peptides only differ in one position: in amD9AKR-12, aspartic acid in position 9 (D9) has been replaced with Ala, increasing the overall cationic charge of the peptide. This substitution has previously been demonstrated to increase the antibacterial activity of KR-12.^21^ Since amKR-12 and amD9AKR-12 both contain two Lys residues that can bind to c-CNF via amidation, this method does not allow the precise control of the peptide orientation in the conjugate. Nevertheless, this synthetic route has fewer steps and does not generate reactive intermediates or otherwise modify the peptide. MD simulations were performed to investigate the amidation reaction. Both Lys residues (K1 and K8) can be conjugated to c-CNF. However, examination of solvent-accessible surface area (SASA, %) revealed that K1 is, on average, more exposed (68% for amKR-12) than K8 (54%). This suggests a preference for K1 to bind to the c-CNF (see Figure S3).

Next, two selective conjugation reactions were explored in order to control the orientation of the peptide during immobilization. In the first approach, a 4-amino acid linker containing an N-terminal cysteine was incorporated into the KR-12 sequence (cysKR-12), with the cysteine thiol group reacting via thiol-ene chemistry with a maleimide previously incorporated onto the c-CNF (Maleimide-CNF). This approach increased the selectivity of the reaction while retaining the Lys amines. In the second approach, an azide group was incorporated into the KR-12 peptide (N_3_KR-12), which could selectively bind to the alkyne in propargyl-CNF via CuAAC.

Interestingly, all three chemical approaches resulted in similar degrees of CNF substitution, with values around 0.18 ± 0.01 mmol peptide/g CNF (Table 1). All materials displayed a homogeneous peptide distribution on the CNF surface when analyzed by scanning electron microscopy with energy-dispersive spectrometry (SEM-EDS) (Figure S4).

### 3.2 Bioactivity Studies

#### 3.2.1 Antibacterial Properties of KR12-CNF Materials

To evaluate the antibacterial activity of the KR12-CNF materials, *E. coli* and *S. aureus* were chosen as representative species of bacteria found in chronic wounds.^40^

**Figure 2:**
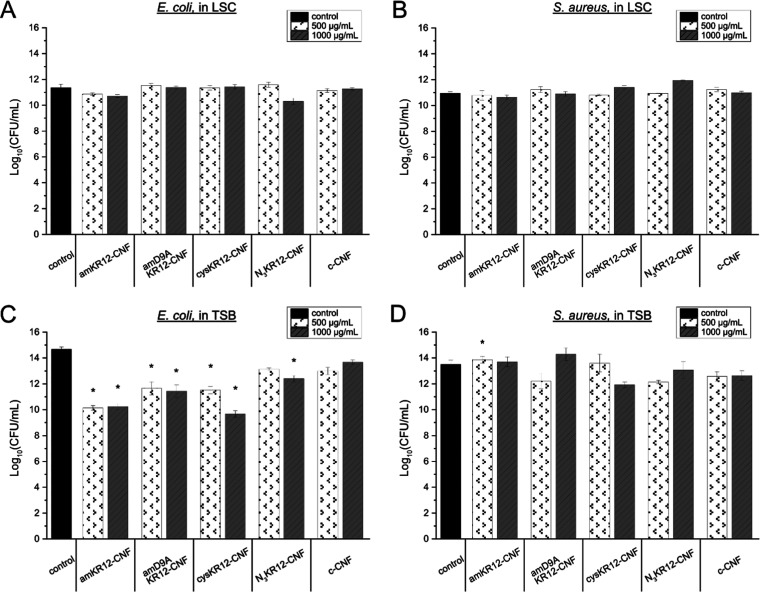
Antibacterial properties of the different KR12-CNF conjugates against *E. coli* and *S. aureus* bacteria. Counts (log CFU/mL) of *E. coli* (A) and *S. aureus* (B) after 1 h in LSC, 2.5 h in TSB for *E. coli* (C), and 4 h in TSB for *S. aureus* (D). Data are expressed as the mean ± standard error of the mean of at least three independent experiments, with statistically significant differences against each c-CNF concentration marked with * (*p* < 0.05). *E. coli* CFU counts (in TSB conditions) found for the KR12-CNF conjugates and c-CNF were significantly different from the CFU counts of the control (*p* < 0.05).

The antibacterial properties of the KR12-CNF materials toward *E. coli* were evaluated after 1 h of exposure to the materials in LSC conditions (Tris·HCl buffer), with results showing no difference between cell numbers for samples incubated with the KR12-CNF conjugates and those that were not exposed (Figure 2A). The bacterial culture was resumed in the presence of TSB and bacterial growth was evaluated by measuring the OD600 and by determining CFUs.

OD600 measurements showed that the growth of *E. coli* exposed to the KR12-CNF materials exhibited concentration-dependent inhibition, reaching a reduction of 60–75%, with respect to the negative control, when exposed to the highest concentrations (500 and 1000 μg/mL) of amKR12-CNF, cysKR12-CNF, and N_3_KR12-CNF (Figure S5). This inhibition of the bacterial growth was significantly larger than the reduction caused by c-CNF alone (40%), which did not show any concentration dependence. The cell viability was evaluated by CFU counting after 2.5 h of exposure to the materials at the two highest concentrations (500 and 1000 μg/mL). These tests confirmed the antibacterial properties of the CNF materials since a significant decrease in the number of viable cells was observed compared to the control (Figure 2C). It can therefore be concluded that the presence of the peptide in the KR12-CNF conjugates significantly increased the antibacterial properties of c-CNF. Among the conjugates, cysKR12-CNF caused the largest decrease in CFUs, with a CFU/mL value 5 log lower than the control.

Free HDPs usually present lower antibacterial activity in bacterial broth than in low salt concentration media.^41^ Notably, the immobilized KR-12 derivatives tested in this project showed the opposite behavior. This lack of activity in LSC could be attributed to a longer time required for the KR12-CNF materials to interact with the bacteria, compared to the free peptides. The difference in activity could also be a consequence of changes in the accessibility and conformation of the immobilized peptide in different experimental conditions (LSC vs TSB). Interestingly, the results indicated that the immobilized peptides were active in TSB, an experimental condition that is usually associated with inhibition of the peptide antibacterial activity.^42^ It should be noted that the free peptides in LSC solutions killed all of the bacteria (results not shown). It is challenging to relate the activity of immobilized HDPs with the performance of the free peptides since the concentration in solution and the peptide density on the surface of CNFs are not directly comparable. Nevertheless, evaluating the activity of the free peptides at concentrations equivalent to the peptide content in the conjugates served as a benchmark for the peptide activities and could be used to detect potential increases in peptide stability caused by immobilization.

The growth curves of *S. aureus* exposed to KR12-CNF materials were highly variable, which made it difficult to evaluate the changes recorded by measuring the OD600. This variability between experiments and the increase in OD600 signals with increased material concentrations (Figure S5) might be explained by the fact that some Gram-positive bacteria, such as *S. aureus*, reversibly aggregate in different media.^43−46^ The CFU count of *S. aureus* exposed to KR12-CNF suspensions was neither significantly different to the negative controls after 1 h of incubation in LSC nor after 4 h in TSB (Figure 2B,D). This indicates that the KR12-CNF suspensions possess no antibacterial properties against *S. aureus* under the conditions tested. The rational for this may be a detrimental effect of the peptide immobilization on the antibacterial activity, combined with the previously reported higher minimum inhibitory concentration (MIC) of KR-12 toward *S. aureus* than to *E. coli* (e.g., 2.5 μM for *E. coli* and 10 μM for *S. aureus* reported by Gunasekera et al. and 2 μM for *E. coli* and 4 μM for *S. aureus* reported by Jacob et al.).^21,23^

#### 3.2.2 Macrophage Response to KR12-CNF Materials

**Figure 3:**
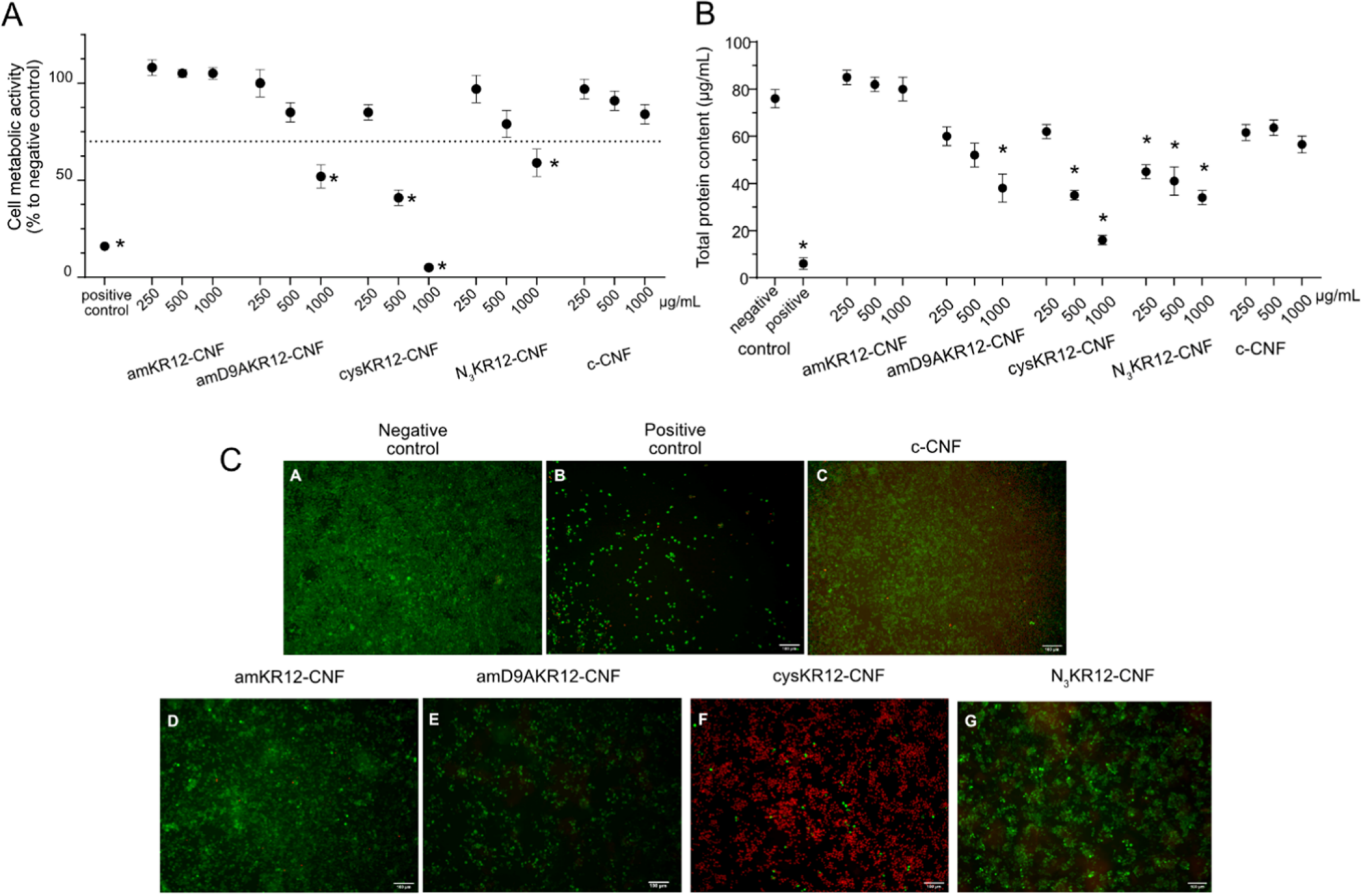
Evaluation of the cytotoxicity of the different KR12-CNF conjugates toward RAW 254.7 cells. The negative control was nonexposed cells and the positive control corresponds to cells exposed to 2.5% DMSO. (A) Metabolic activity of RAW 254.7 cells after 24 ± 2 h of exposure to the KR12-CNF materials (concentration range 250–1000 μg/mL) expressed as a percentage of the negative control ± standard error of the mean of at least three independent experiments. Significant differences to the 70% cytotoxic limit are marked with * (*p* < 0.05). (B) Total protein content in the lysate of RAW 254.7 cells after 24 ± 2 h of exposure to the materials (concentration range 250–1000 μg/mL). Significant variations from the negative control are marked with * (*p* < 0.05). (C) Representative images of live/dead stained RAW 254.7 cells after 24 ± 2 h of exposure to 1000 μg/mL KR12-CNF suspensions. Viable cells appear green and cells with compromised membrane integrity appear red. The scale bar represents 100 μm.

Macrophages play a central role in the wound-healing process, and it was therefore prudent to assess the cytotoxicity and capacity of the KR12-CNF conjugates to regulate the macrophage response to LPS stimulation. The cytotoxicity tests revealed that cysKR12-CNF was the most cytotoxic in all of the measurements investigated (cell metabolic response, total protein content, and cell membrane integrity). This was followed by amD9AKR12-CNF and N_3_KR12-CNF, while amKR12-CNF did not exhibit any cytotoxic effects under the conditions of the study (Figure 3). The starting material c-CNF was not cytotoxic, in agreement with previous publications.^47−49^ It is noted that the intermediate products maleimide-CNF and propargyl-CNF (see reaction schemes in Figure 1) were not found to be cytotoxic either (Figure S6).

**Figure 4:**
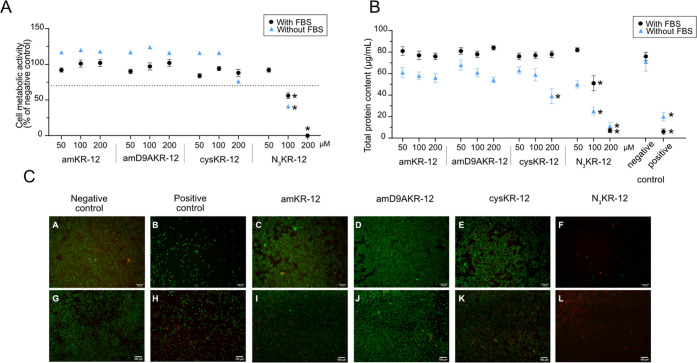
Evaluation of the cytotoxicity of the KR-12 derivatives toward RAW 254.7 cells in the presence and absence of serum in the cell culture medium (with FBS and without FBS, respectively). Negative control corresponds to nonexposed cells and positive control corresponds to cells exposed to 2.5% DMSO. (A) Cell metabolic activity of RAW 254.7 cells after 24 ± 2 h of exposure to the materials (concentration range 50–200 μM) expressed as the percentage to the negative control ± standard error of the mean of at least three independent experiments. Significant difference to the 70% cytotoxic limit is marked with * (*p* < 0.05). (B) Total protein content evaluated in the lysate of RAW 254.7 cells after 24 ± 2 h of exposure to the materials (concentration range 50–200 μM). Significant results as compared to the negative control are marked with * (*p* < 0.05). (C) Representative live/dead staining images of RAW 254.7 cells after 24 ± 2 h exposure to the KR-12 derivatives at 200 μM in the presence of serum (upper panels, A–F) and in the absence of serum (lower panels, G–L). Viable cells appear green and cells with compromised membrane integrity appear red. The scale bar represents 100 μm.

The cytotoxicity of the free KR-12 derivatives was also evaluated at concentrations equivalent to the peptide content in the KR12-CNF materials. The results showed that amKR-12, amD9AKR-12, and cysKR-12 were not cytotoxic; however, macrophages exposed to N_3_KR-12 showed low cell metabolic activity and almost no cell survival at the highest concentration tested (200 μM) (Figure 4). The cytotoxicity of the free N_3_KR-12 was attributed to its azide group at the N-terminus.^50^ When the peptide is immobilized onto the CNFs, the azide group is consumed in the azide-alkyne cycloaddition between the propargyl-CNF and the N_3_KR-12 peptide; see Figure 1. Therefore, the cytotoxicity of N_3_KR12-CNF cannot be attributed to this azide group.

The free peptides did not exhibit any cytotoxic effects, but high concentrations of the conjugates significantly affected the viability of the cells. It was therefore hypothesized that peptide immobilization may have improved the peptide activity (i.e., cytotoxicity) in serum, a condition where the activity of free peptides is usually inhibited.^51,52^ To evaluate this hypothesis, the cytotoxicity of the free peptides and the KR12-CNF materials was evaluated in serum-free cell media. The macrophages exposed to the free peptides in serum-free media showed slightly higher metabolic activity but lower cell numbers (i.e., lower protein content and a lower ratio of viable to nonviable cells) than macrophages exposed to the peptides in the serum-supplemented cell media (Figure 4). Signs of toxicity appeared in the absence of the serum, with cysKR-12 being more cytotoxic than amD9AKR-12 and amKR-12 in the serum-free media. The cytotoxic effect of the KR12-CNF materials in serum-free conditions was similar or slightly higher than that observed in the serum-supplemented media (Figure S7). Altogether, these results indicate that the cytotoxic activity of the KR-12 derivatives toward macrophages was not significantly affected by the presence of the serum when the peptides were immobilized onto CNF. However, the free peptides, classified as noncytotoxic when tested in the serum-supplemented media, showed certain signs of cytotoxicity in serum-free conditions.

**Figure 5:**
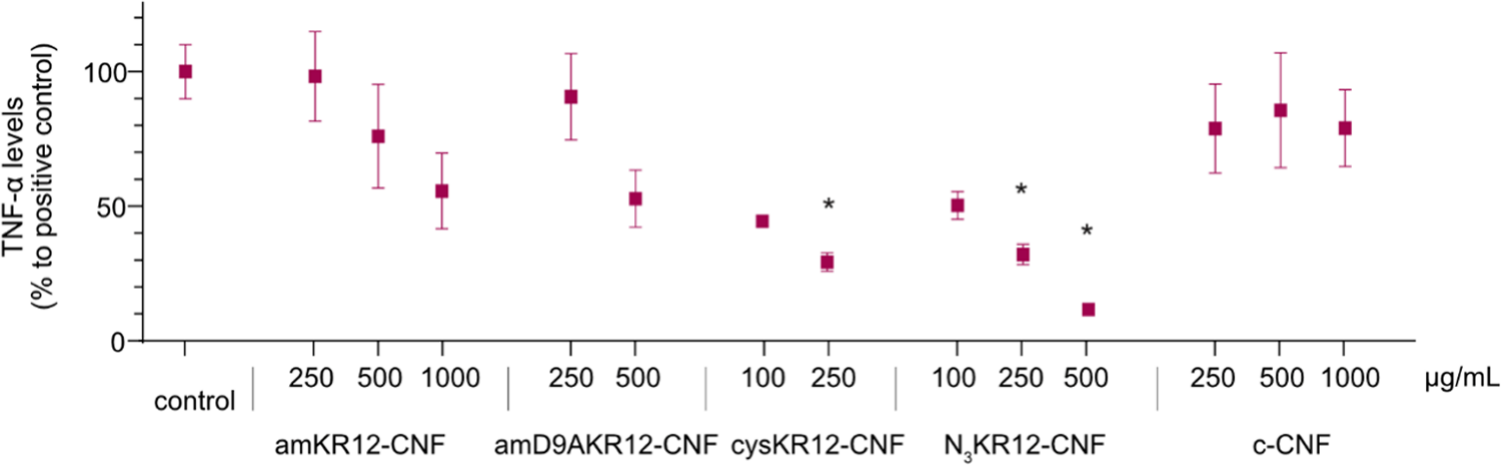
TNF-α levels secreted by RAW 264.7 cells after 24 ± 2 h stimulation with 10 ng/mL LPS and simultaneous exposure to suspensions of the KR12-CNF materials (the concentration range was selected according to the cytotoxicity results). The results were normalized to the protein content and expressed as a percentage of the positive control (LPS stimulation alone). Data corresponds to the mean of five independent experiments ± standard error of the mean. Statistically significant differences to c-CNF are marked with * (*p* < 0.05).

The concentrations that did not cause a cytotoxic effect were selected to evaluate the capacity of different KR12-CNF materials to modulate the response of macrophages to LPS stimulation. The levels of the proinflammatory cytokine TNF-α were evaluated after 24 ± 2 h of LPS-induced inflammation, and the results showed a concentration-dependent decrease in TNF-α levels when the cells were exposed to the KR12-CNF conjugates. The TNF-α concentration decreased by 90%, with respect to the positive control (LPS stimulation alone), when the cells were treated with 500 μg/mL of N_3_KR12-CNF and 70% when exposed to 250 μg/mL cysKR12-CNF. Both 500 μg/mL amD9KR12-CNF and 1000 μg/mL amKR12-CNF conjugates caused a 50% relative reduction in the TNF-α levels (Figure 5). The c-CNF alone did not inhibit the secretion of TNF-α in a concentration-dependent fashion; instead, a constant inhibition of 40% was observed (Figure 5). The inhibitory effect of c-CNF may be caused by the entrapment of LPS within the cellulose nanofibers. Nevertheless, the peptide conjugation had a significant effect on the capacity of the CNFs to modulate TNF-α secretion, especially when the conjugation took place via selective chemistry (N_3_KR12-CNF and cysKR12-CNF). When comparing the inhibitory effect of the conjugates with the activity of the free peptides, it was found that cysKR12-CNF was more active than the free cysKR12, while the opposite was found for the other KR-12 derivatives and their conjugates (Figure S8). The levels of TNF-α normalized to protein content as well as the effect of the materials alone on TNF-α secretion by macrophages can be found in Figure S8.

### 3.3 MD Simulations of KR12-CNF Materials

MD simulations were applied to investigate how different immobilization approaches and peptide designs affected the accessibility and conformation of the KR-12 peptides. Both SASA and the secondary structures were calculated theoretically and compared to the free amKR-12, N_3_KR-12, and cysKR-12 peptides. In every case, the models contained two peptides attached to the cellulose. The secondary structures calculated were slightly different between each of the conjugates and the corresponding free peptides (Figure S9). The results suggest that immobilized peptides still retain their secondary structures and may, therefore, retain their bioactivity. One of the cysKR-12 peptides anchored into cellulose was found to form a characteristic α-helix in the simulations, and this differs from the other KR-12 derivatives that almost exclusively formed loops. This difference in the secondary structure for this particular cysKR12-CNF conjugate might be responsible for its enhanced bioactivity over the other conjugates.^15^

The dynamic presentation of peptides immobilized on CNF was evaluated by calculating the SASA (%) values. For all models tested, the values were in the range of 60–80%, using the corresponding free peptides as references. Although these peptides are exposed enough, the SASA values were always smaller than those of the free peptides, indicating that immobilization onto CNF decreases the accessibility of the peptides. Of the conjugates investigated, cysKR12-CNF proved to be the most accessible (77%), followed by N_3_KR12-CNF with a SASA value of around 71%, reinforcing the bioactivity of these materials.

In summary, cysKR12-CNF was the conjugate that stood out in all of the tests performed. In this conjugate, peptide immobilization took place via a selective conjugation that allowed the peptide orientation to be controlled. The peptides reacted with the CNFs via their cysteine thiol group, located at the N-terminus, far away from the hydrophobic residue located at the C-terminus. As stated by Song et al., this strategy considered the fact that the hydrophobic residue of HDP is fundamental for its insertion into the bacterial cell membrane.^53^ Therefore, keeping this residue as far away from the immobilization site is believed to help retain the antibacterial activity of the immobilized peptide. Furthermore, this conjugation does not involve the amine groups of Lys, which also play a key role in the bioactivity of KR-12.^21,54^ The same strategy was applied in the synthesis of N_3_KR12-CNF, albeit exploiting different reactive groups. However, in this instance, the conjugate was found to be less efficient in killing bacteria as well as less cytotoxic to macrophage cells than cysKR12-CNF. Both selective chemistries tested, thiol-ene and CuAAC click chemistries, successfully conjugated spacers onto the CNFs to which the peptides could be attached. It has been suggested that longer spacers could enhance the antimicrobial activity of immobilized peptides as they provide flexibility to the interaction between the peptide and the cell membrane.^15,55^ When comparing cysKR12-CNF with N_3_KR12-CNF, the cysKR12-CNF had a longer spacer and a 4-amino acid linker, which could account for its increased activity. The outcomes of the MD simulations support the hypothesis described above, with cysKR12-CNF being the only conjugate whose peptide displayed a well-established secondary structure and was the most accessible.

It is interesting to note that while the cytotoxicity and antibacterial activity of N_3_KR12-CNF and cysKR12-CNF were different, their inhibition of TNF-α secretion was similar. This could suggest that the mechanism behind LPS neutralization by the peptides may be less demanding in terms of peptide exposure and conformation. This process is less studied than the antibacterial mechanism of HDPs. Studies have suggested that HDP strong binding to LPS is not enough to neutralize LPS-induced inflammation. Other mechanisms of LPS neutralization involve the dissociation of LPS aggregates, which are believed to be the biologically active form of LPS. Alternatively, cathelicidine-HDPs were found to compete with LPS to bind to macrophages.^22,56^ In any case, it has been suggested that the peptide net charge, hydrophobicity, amphiphilicity, and secondary structure impact the mechanism and efficacy of the HDP interaction with LPS.^22,56^

When evaluating the effect of amino acid substitutions on the bioactivity of the conjugates, it was found that the previously reported higher bactericidal potency of D9AKR-12 than KR-12^21^ was lost after peptide immobilization. Gao et al. reported that the mechanism by which the immobilized peptides kill bacteria might be different from that of the free peptides,^57^ which could explain this finding. Interestingly, both free peptides showed similar toxicities toward macrophages, while when immobilized, amD9AKR-12 was more cytotoxic than amKR-12 in both serum and serum-free conditions.

Another interesting finding of this work was the cytotoxic effects of amD9AKR12-CNF and cysKR12-CNF conjugates, given that the free peptides and c-CNF are not cytotoxic. This implies that the immobilization of peptides onto CNFs protected them from serum components that normally inhibit their function. These findings highlight the importance of performing in vitro studies that mirror physiological conditions as closely as possible.

Regarding the potential use of the KR12-CNF materials in the treatment of chronic wounds, this study has shown that the functionalization of CNFs with KR-12 resulted in bioactive composites with antibacterial and anti-inflammatory properties. The most bioactive conjugate was also cytotoxic at the highest concentrations tested. Nevertheless, these results indicate that it is possible to find a therapeutic window for the safe use of KR12-CNF conjugates as antibacterial and/or anti-inflammatory wound dressings.

## 4 Conclusions

The present work showed that it is possible to endow bioactivity to CNFs by functionalizing the fibers with the HDP KR-12. This represents a step forward in the development of CNF-based materials for the treatment of chronic wounds. The results reflect the challenges of maintaining the peptide activity when immobilized, as well as the difficulty in achieving specificity for bacterial over mammalian cells using active molecules that target the cell membrane. KR-12 conjugation via the thiol-ene reaction yielded the most bioactive KR12-CNF composite, a finding than can be attributed to the peptide conformation and accessibility, controlled by the selectivity of the immobilization chemistry and length of the linker in the conjugate.

## Supporting Information Available

Methods for SEM/EDS and MD simulations analysis, sequence of the KR-12 derivatives under study (Figure S1); HPLC analytical trace and LCMS mass identity of the synthesized peptides (Figure S2); root-mean square deviation (RMSD) and SASA% simulations for free peptides (Figure S3); representative images of the scanning of carbon, oxygen, and nitrogen by SEM-EDS for the KR12-CNF conjugates (Figure S4); bacterial growth at the exponential phase expressed as percentage of the negative control (OD600 measurements) for gram-negative *E. coli* and gram-positive *S. aureus* bacteria exposed to the KR12-CNF materials (Figure S5); evaluation of the cytotoxicity of the intermediate species maleimide-CNF and propargyl-CNF toward RAW 254.7 cells (Figure S6); evaluation of the cytotoxicity of the KR-12 derivatives toward RAW 254.7 cells in the presence and absence of serum in the cell culture medium (Figure S7); TNF-α levels secreted by RAW 264.7 cells after 24 h stimulation with 10 ng/mL LPS and exposure to KR12-CNF materials (A) and soluble KR-12 derivatives (B) (Figure S8); and RMSD and SASA% plots along the simulation time for the KR12 conjugates (Figure S9) (PDF)
