# Supplementary material for: KR-12 Derivatives Endow Nanocellulose with Antibacterial and Anti-Inflammatory Properties: Role of Conjugation Chemistry
Source: ACS Appl Mater Interfaces. 2023 May 11;15(20):24186–96. doi: 10.1021/acsami.3c04237 (PMC10214380; doi:10.1021/acsami.3c04237)
Supplement: Supplementary file 1 — am3c04237_si_001.pdf [file am3c04237_si_001.pdf]

Supporting Information

KR-12 derivatives endow nanocellulose with antibacterial and anti-inflammatory properties: Role of conjugation chemistry

Anna Blasi-Romero [1], Molly Ångström [1], Antonio Franconetti [2], Taj Muhammad [3], Jesús Jiménez-Barbero [2,4,5,6] Ulf Göransson [3], Carlos Palo-Nieto [1], Natalia Ferraz [1*]*

[1] Division of Nanotechnology and Functional Materials, Department of Materials Science and Engineering, Uppsala University, Box 35, SE-75103, Uppsala, Sweden.

[2] CIC bioGUNE, Derio-Bizkaia 48160, Spain.

[3] Pharmacognosy, Department of Pharmaceutical Biosciences, Biomedical Centre, Uppsala University, Box 591, SE-75124, Uppsala, Sweden.

[4] Department of Inorganic & Organic Chemistry, Faculty of Science and Technology, University of the Basque Country, Leioa 48940, Spain.

[5] IKERBASQUE, Basque Foundation for Science and Technology, Bilbao 48009, Spain.

[6] Centro de Investigacion Biomedica En Red de Enfermedades Respiratorias, Madrid 28029, Spain.

*Shared last authorship, corresponding authors: carlos.nieto@angstrom.uu.se, natalia.ferraz@angstrom.uu.se

1. Methods

1.1. Scanning Electron Microscopy/Energy Dispersive Spectroscopy (SEM/EDS)

The distribution of the KR-12 derivatives on the KR12-CNF materials was investigated using scanning electron microscopy (SEM) imaging together with energy dispersive spectroscopy to detect nitrogen. The KR12-CNF suspensions were air-dried, placed on a carbon stub, and coated with a thin layer of gold/palladium with a sputter coater Polaron SC7640 sputter coater (Thermo VG Scientific) to be imaged using a Zeiss LEO 1550 SEM with SE2 detector and an energy dispersive detector EDS (Carl Zeiss Microscopy, Oberkochen Germany).

1.2. *Molecular Dynamics Simulations: Model and simulation*

System preparation. To prepare the linkers for molecular dynamics simulations, models (maleimide, triazole and carboxymethyl units) were firstly built by GaussView software and optimized using the Gaussian 16 package.[1] Frequency analyses were also performed to ensure that each structure was a true minimum ($N_{imag} = 0$). Then, the conformational space was evaluated for each structure using the MacroModel module included in the Maestro software.[2] For this purpose, the MMFFs force field [3] was applied including water as solvent. An extended level of torsion sample options was selected and a maximum of 20 structures were saved for each fragment. Each conformation was subsequently optimized at B3LYP or HF/6-31G* level of theory. Diffuse functions were employed for charged fragments. This procedure allows to overcome issues about atomic partial charges associated to conformation dependency. Two different schemes were accounted to be consistent with AMBER and GLYCAM [4] parametrizations. For the carboxymethyl unit, the employed scheme involves the calculation of the atomic partial charges at HF/6-31G* level and subsequent one-stage RESP fitting (CHELPG method) with a charge

restraint weight of 0.01 thus making equivalent the aliphatic protons. For triazole and maleimide fragments, a two-stage fitting was employed also assigning the same partial charge on aliphatic protons (atomic charge equilibration predicted by atom paths). These fitting procedures were carried out based on LIBRETA library implemented in Multiwfn software.[5] Finally, parameters for these linkers were generated following an in-house-developed protocol for combining *GLYCAM_06j-1* and *gaff2* force fields in a semiautomatic manner

System equilibration and MD simulations. The Amber20 software[6] with *GLYCAM_06j-1*, *ff14SB*[7] and *gaff2*[8] force field parameters were applied for cellulose, peptides and linkers, respectively. The initial structures were neutralized with either Na^+ or Cl^- ions and set at the center of a cubic TIP3P[9] water box with a buffering distance between the solute and box of 10 Å. Three steepest descent/conjugate gradient minimizations (maximum 5000 steps) were carried out on the initial structures of all systems. In the first minimization, the positions of cellulose, peptides and ions were restrained using Harmonic potential restraints with a force constant k of 100 kcal mol^{-1} Å^{-2}. Subsequent minimizations were performed without any restraints. After minimization, the system was gradually heated from 0 to 300 K during 100 ps (NVT assemble) under the Andersen temperature coupling[10] scheme. Assuming periodic boundary conditions (PBC), the particle mesh Ewald method was employed for modelling long-range electrostatic interactions whereas the cut-off for non-bonded interactions was set to 8 Å.[11] The SHAKE algorithm[12] was also used to constraint covalent bonds involving hydrogen atoms. Then, the system was equilibrated for 2 ns at a constant volume and temperature of 300 K. The final snapshot of this equilibration was

used as jumping-off point for MD production under the same conditions. Two consecutive MD simulations (100 ns each) was run to obtain the final production trajectory. Three independent replicas were carried out for each system.

Data analysis. Solvent Accessible Surface Area (SASA) was calculated along the simulation by using the *cpptraj* module.[13] Raw data were referenced to the maximum value (in Å^2) obtained for the free ligand in a water box (100% SASA). Then, a bisquare smoothing algorithm with a sampling proportion of 0.1 was applied for the final plot representation. The secondary structure for each peptide was calculated using the DSSP algorithm[14] as implemented in the *cpptraj* module.

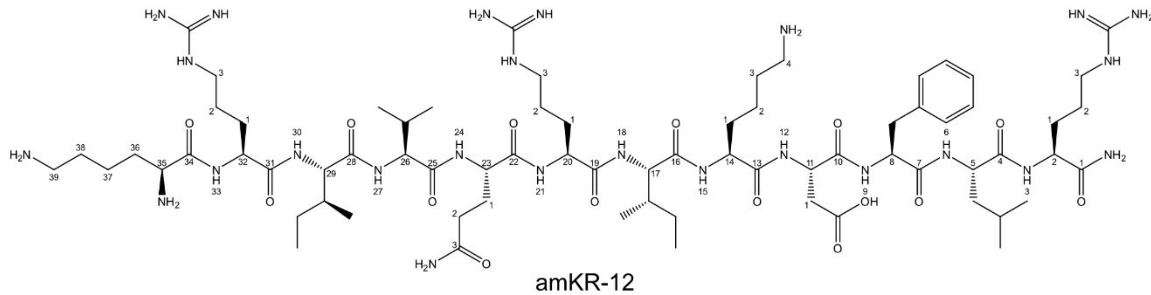

amKR-12

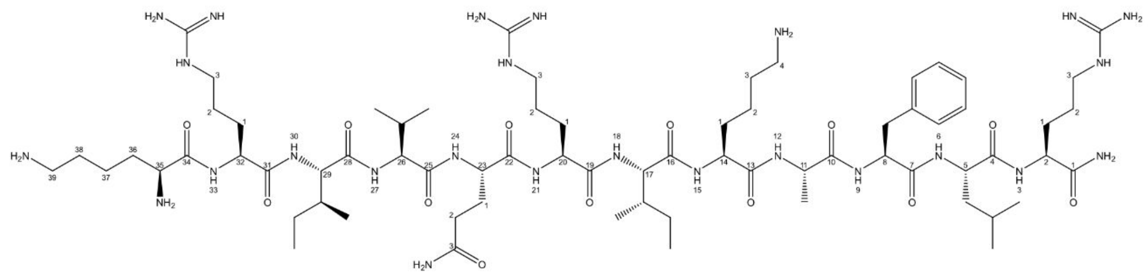

amD9AKR-12

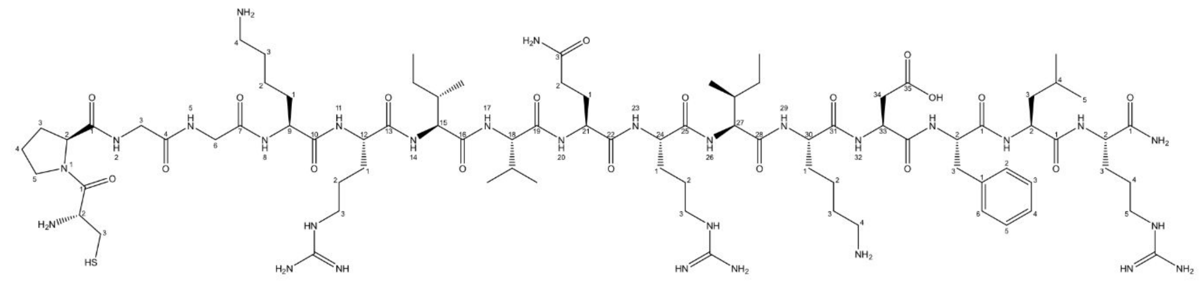

cysKR-12

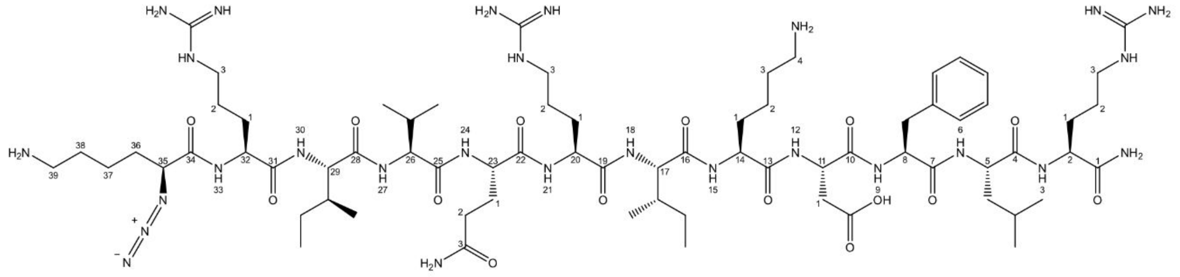

N_3KR-12

Figure S1. Sequences of the KR-12 derivatives under study.

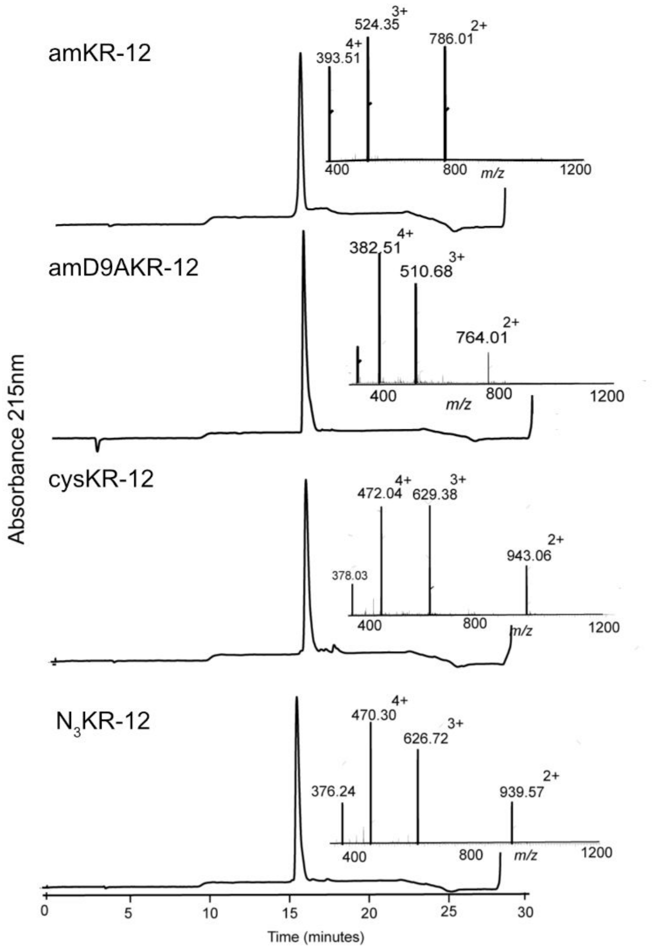

Figure S2. HPLC analytical trace and LC-MS mass identity of the synthesized peptides. A gradient of 5-50% solvent B in 18 minutes (solvent A: 0.1% trifluoroacetic acid (TFA) in water and solvent B: 100% acetonitrile containing 0.1% TFA) was used for analytical RP-HPLC, absorbance measurements at 215 nm.

2. Results

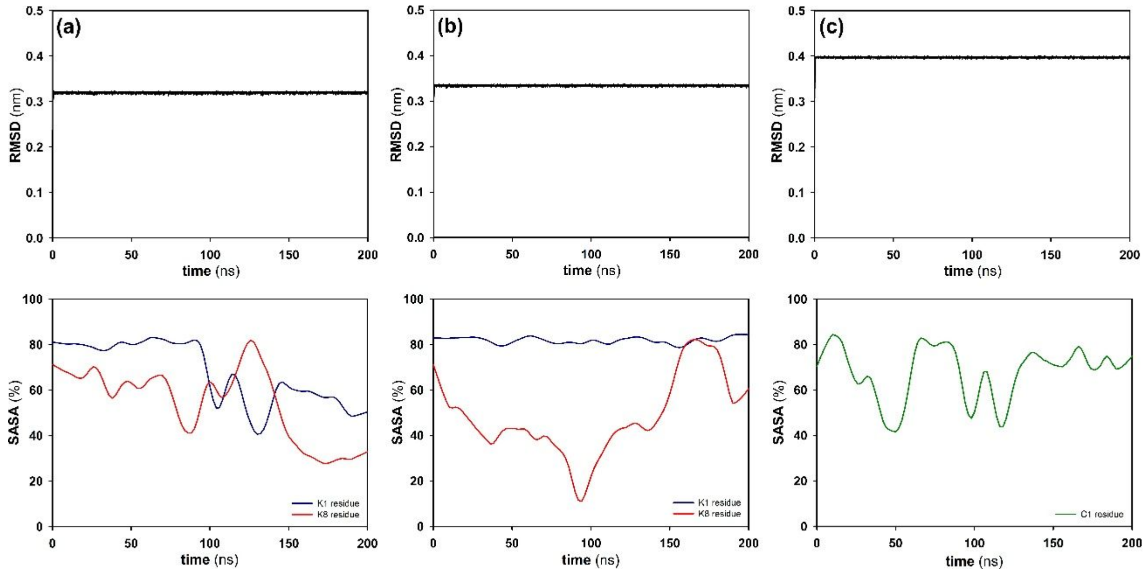

Figure S3. Root-mean-square deviation (RMSD) and solvent accessible surface area (SASA, %) plots along the simulation time for: (a) amKR-12; (b) amD9AKR-12 and (c) cysKR-12 peptides. SASA values were calculated for lysine (K1 and K8) and cysteine residues.

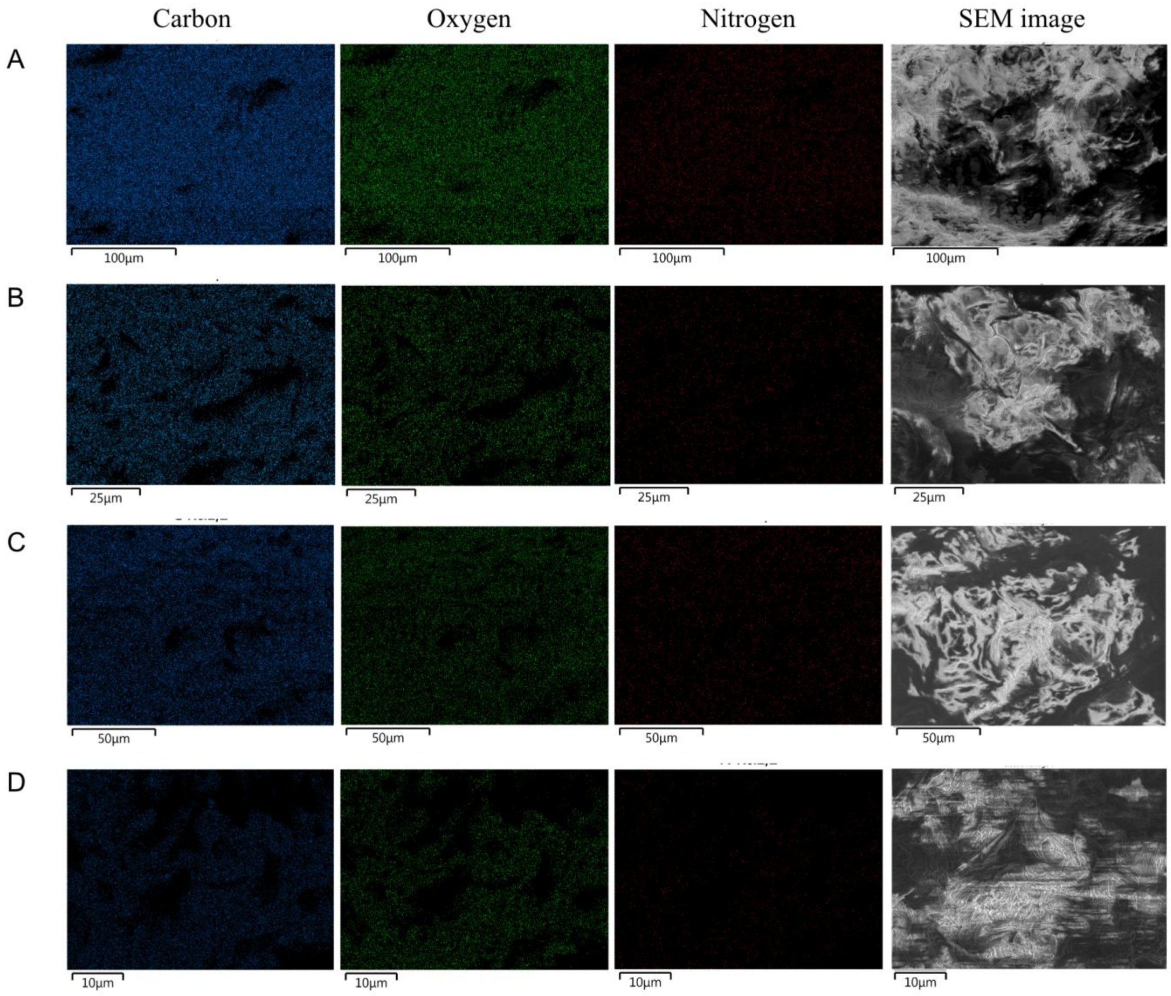

Figure S4. Representative images of the scanning of carbon, oxygen and nitrogen by SEM-EDS of (A) amKR12-CNF, (B) amD9AKR12-CNF, (C) cysKR12-CNF and (D) N_3KR12-CNF.

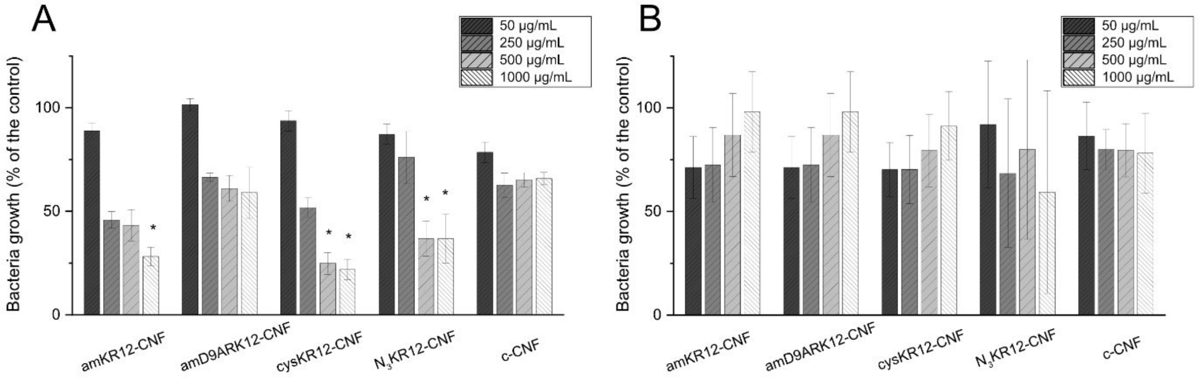

Figure S5: Bacterial growth at the exponential phase (3 h and 5 h for *E. coli* and *S. aureus* respectively) expressed as percentage of the negative control (OD600 measurements) for *E. coli* (A) and *S. aureus* (B) bacteria exposed to the KR12-CNF materials. Data is expressed as the mean ± standard error of the mean of at least three independent experiments, with statistically significant difference against each c-CNF concentration marked with * ($p<0.05$).

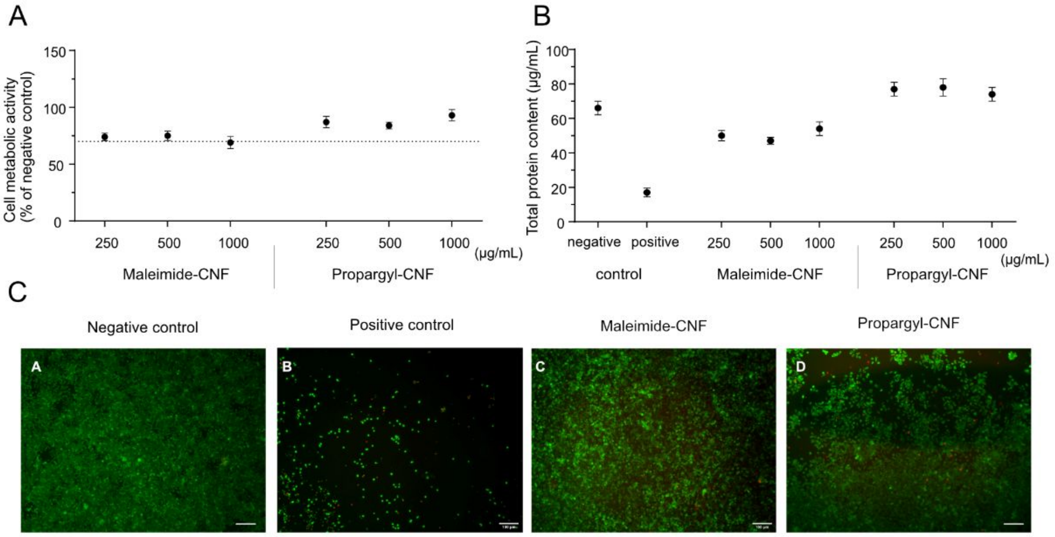

Figure S6. Evaluation of the cytotoxicity of the intermediate species maleimide-CNF and propargyl-CNF towards RAW 254.7 cells. Negative control corresponds to non-exposed cells and positive control corresponds to cells exposed to 2.5% DMSO. (A) Cell metabolic activity of RAW 254.7 cells after 24 ± 2 h of exposure to the materials (concentration range 250-1000 ug/ml) expressed as the percentage to the negative control ± standard error of the mean of at least three independent experiments. No significant difference respect to the 70% cytotoxic limit was found. (B) Total protein content evaluated in lysate of RAW 254.7 cells after 24 ± 2 h of exposure to the materials (concentration range 250-1000 ug/ml). Results were not significantly different from the negative control. (C) Representative images of live/dead staining images of RAW 254.7 cells after 24 ± 2 h exposure to maleimide-CNF and propargyl-CNF suspensions at 1000 ug/ml. Viable cells appear green and cells with compromised membrane integrity appear red. Scale bar represents 100 µm.

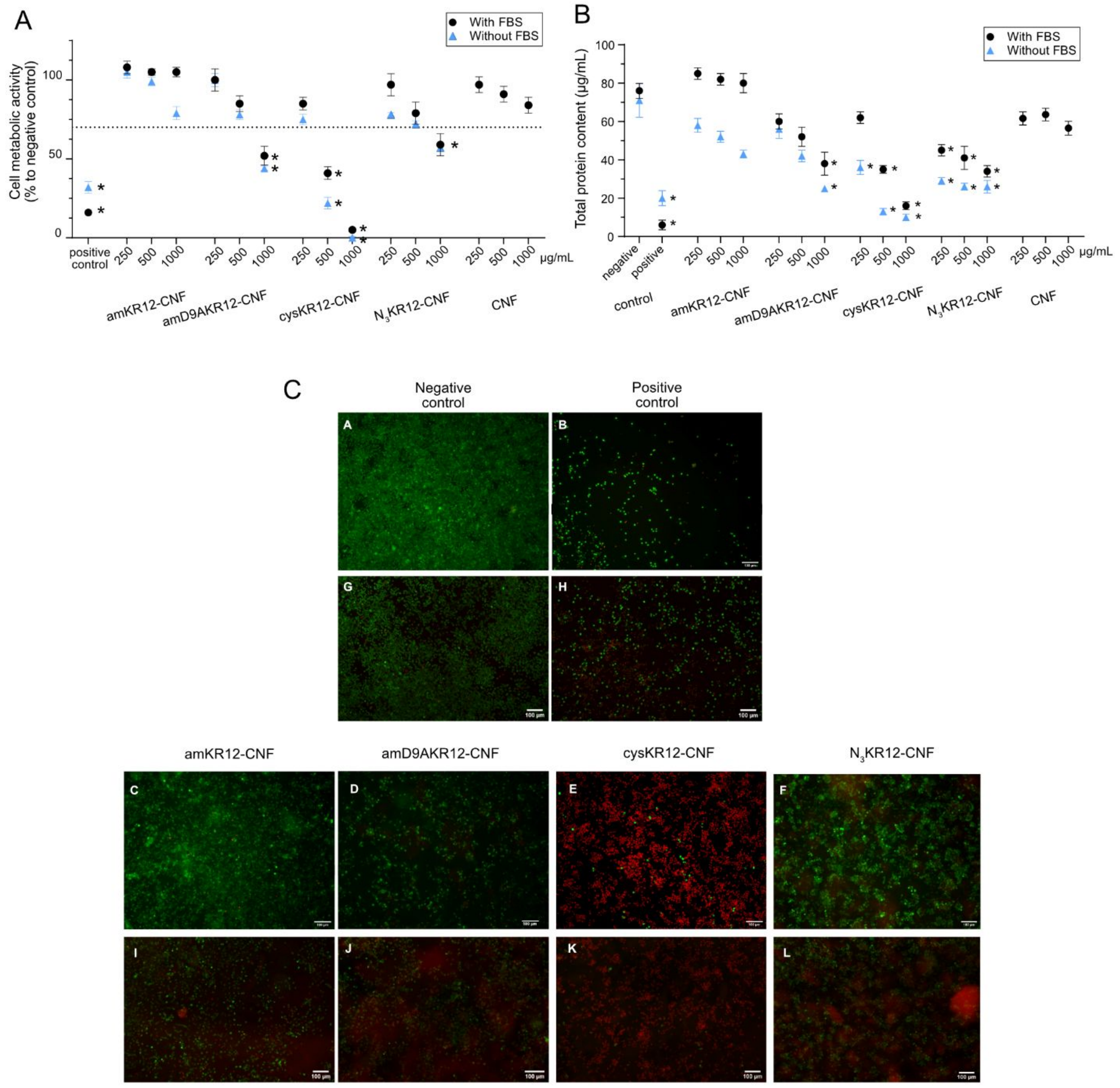

Figure S7. Evaluation of the cytotoxicity of the KR12-CNF materials towards RAW 254.7 cells in the presence and absence of serum in the cell culture medium (with FBS and without FBS, respectively). Negative control corresponds to non-exposed cells and positive control corresponds to cells exposed to 2.5% DMSO. (A) Cell metabolic activity of RAW 254.7 cells after 24 ± 2 h of exposure to the materials (concentration range 250-1000 µg/mL) expressed as the percentage to

the negative control ± standard error of the mean of at least three independent experiments. Significant difference to the 70% cytotoxic limit is marked with * ($p<0.05$). (B) Total protein content evaluated in lysate of RAW 254.7 cells after 24 ± 2 h of exposure to the materials (concentration range 250 -1000 µg/mL). Significant results as compared to the control are marked with * ($p<0.05$). (C) Representative images of live/dead staining images of RAW 254.7 cells after 24 ± 2 h exposure to the KR-12 derivatives at 1000 µg/mL in the presence of serum (upper panels, A to F) and in the absence of serum (lower panels, G to L). Viable cells appear green and cells with compromised membrane integrity appear red. Scale bar represents 100 µm.

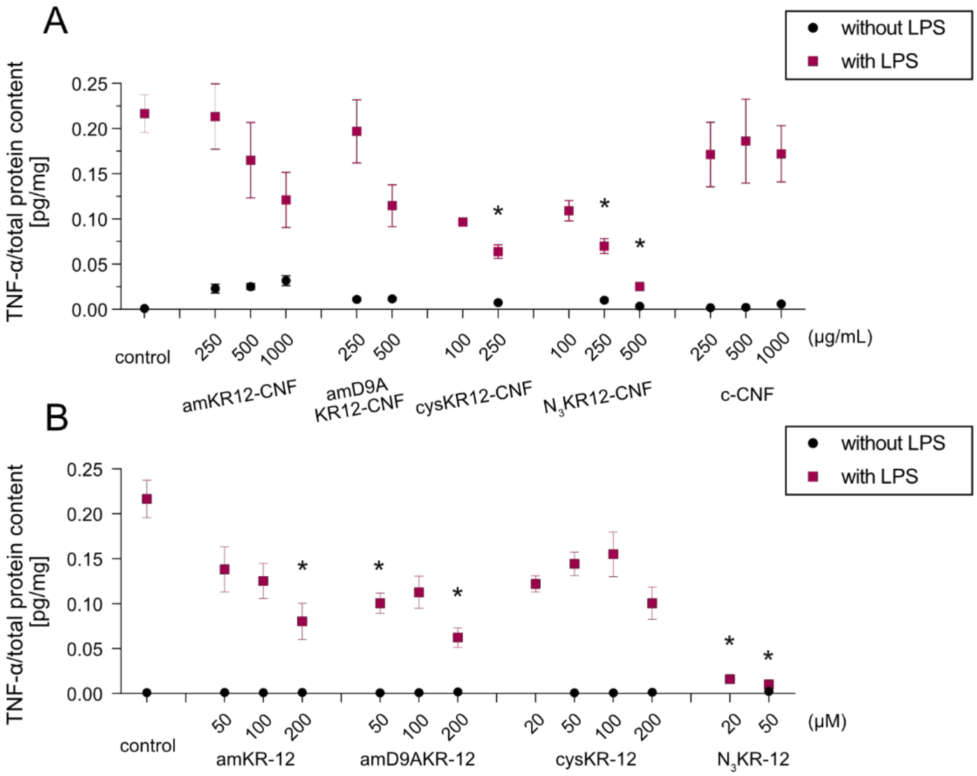

Figure S8. TNF-α levels secreted by RAW 264.7 cells after 24 ± 2 h stimulation with 10 ng/mL LPS and exposure to KR12-CNF materials (A) and soluble KR-12 derivatives (B). Data are normalized to the protein content and presented as the mean of five independent experiments with standard error of the mean. Statistically significant differences to the c-CNF for the same concentration (A) or to the control (B) are marked with * ($p<0.05$). Figures A and B also show the levels of TNF-α levels secreted by RAW 264.7 cells when exposed to the materials (KR12-CNF conjugates and KR-12 derivatives) without LPS stimulation.

(a)

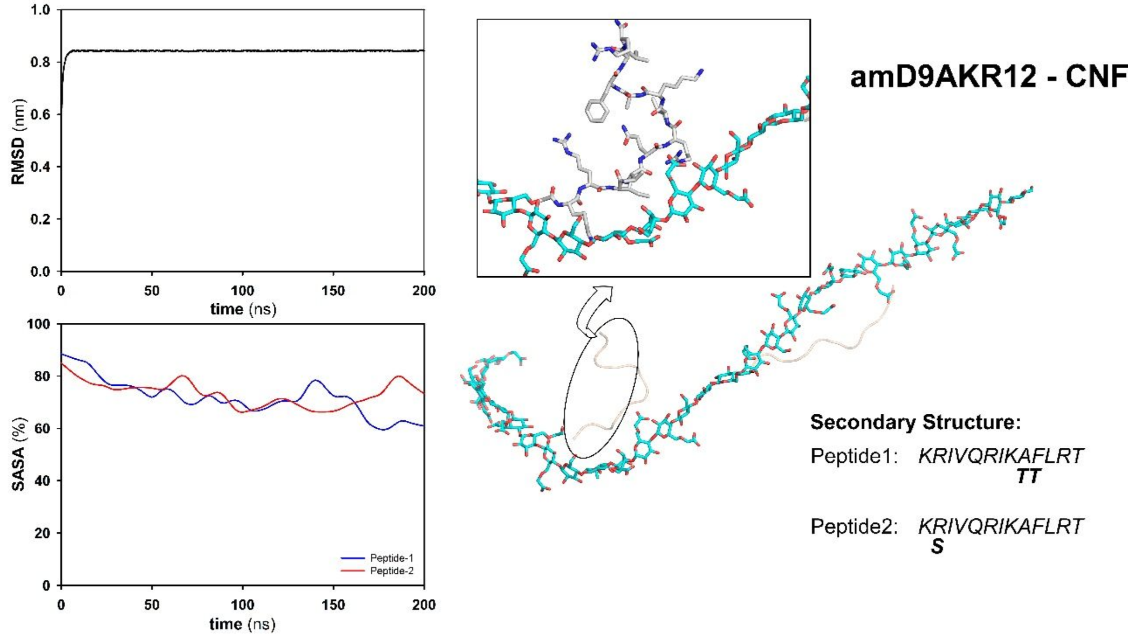

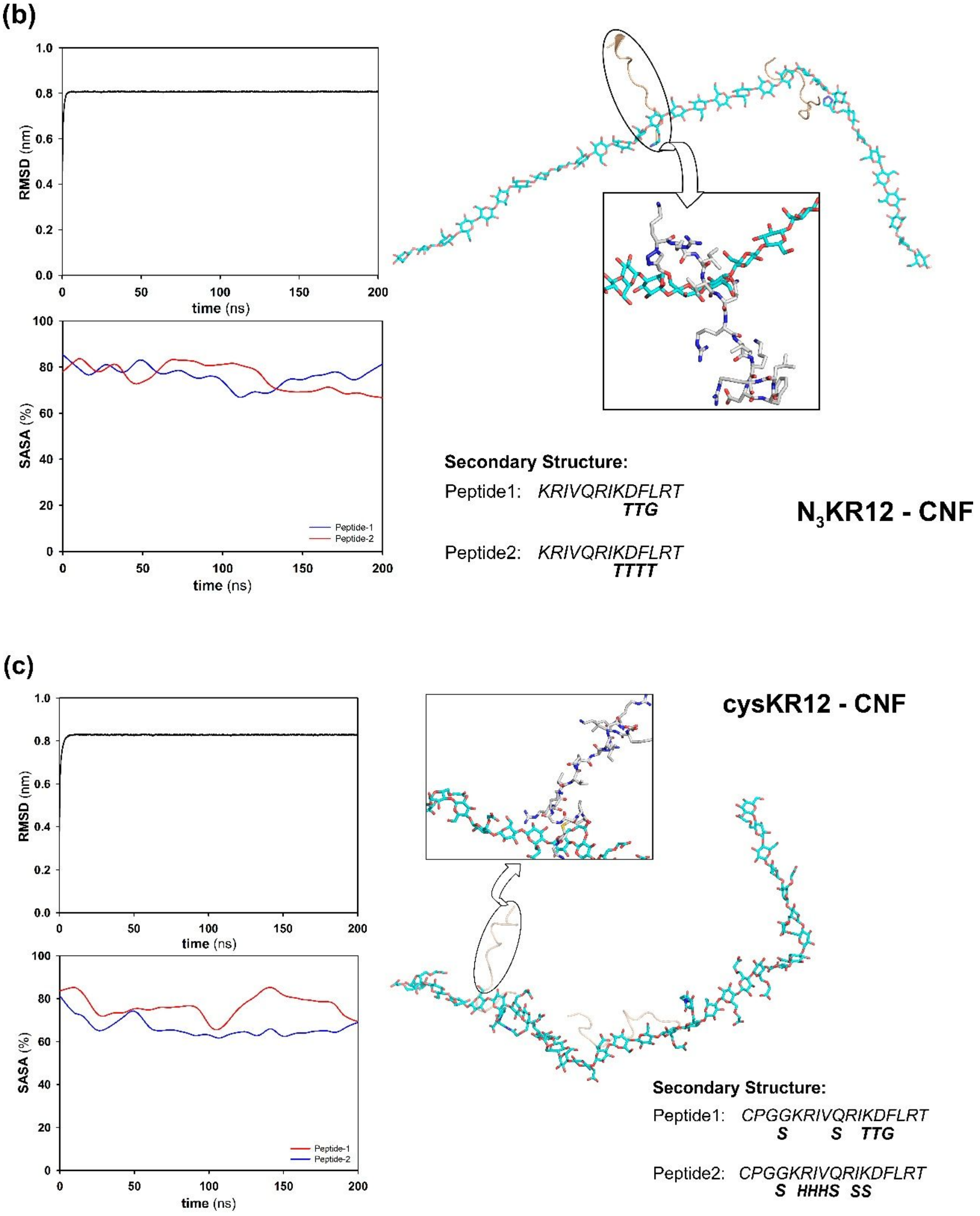

Figure S9. Root-mean-square deviation (RMSD) and solvent accessible surface area (SASA, %) plots along the simulation time for: (a) amD9AKR12–CNF; (b) N_3KR12–CNF and (c) cysKR12–CNF materials. Raw and smoothed data were shown for each model. Snapshots were also

displayed. The calculated secondary structure for each material was classified based on the DSSP algorithm and grouped into classes: helix (G, H and I), strand (E and B) and loop (S, T, and C, whereas C is represented as blank space). Letter codes: “G”, 3-10 helix; “H”, alpha helix; “I”, π-helix; “E”, β-sheet; “B”, β-bridge; “S”, bend; “T”, helix turn and “C”, coil.

References

1. M. J. Frisch, G. W. T., H. B. Schlegel, G. E. Scuseria, M. A. Robb, J. R. Cheeseman, G. Scalmani, V. Barone, G. A. Petersson, H. Nakatsuji, X. Li, M. Caricato, A. Marenich, J. Bloino, B. G. Janesko, R. Gomperts, B. Mennucci, H. P. Hratchian, J. V. Ortiz, A. F. Izmaylov, J. L. Sonnenberg, D. Williams-Young, F. Ding, F. Lipparini, F. Egidi, J. Goings, B. Peng, A. Petrone, T. Henderson, D. Ranasinghe, V. G. Zakrzewski, J. Gao, N. Rega, G. Zheng, W. Liang, M. Hada, M. Ehara, K. Toyota, R. Fukuda, J. Hasegawa, M. Ishida, T. Nakajima, Y. Honda, O. Kitao, H. Nakai, T. Vreven, K. Throssell, J. A. Montgomery, Jr., J. E. Peralta, F. Ogliaro, M. Bearpark, J. J. Heyd, E. Brothers, K. N. Kudin, V. N. Staroverov, T. Keith, R. Kobayashi, J. Normand, K. Raghavachari, A. Rendell, J. C. Burant, S. S. Iyengar, J. Tomasi, M. Cossi, J. M. Millam, M. Klene, C. Adamo, R. Cammi, J. W. Ochterski, R. L. Martin, K. Morokuma, O. Farkas, J. B. Foresman, and D. J. Fox. "Gaussian 09, revision a.01 " *Inc., Wallingford CT* (2016)
2. Schrödinger Release 2023-1:Macromodel, schrödinger., LLC, New York, NY (2021)
3. Halgren, T. A. "Mmff vi. Mmff94s option for energy minimization studies." *Journal of Computational Chemistry* (1999): 20, 720-29. Doi 10.1002/(Sici)1096-987x(199905)20:7<720::Aid-Jcc7>3.0.Co;2-X.
4. Kirschner, K. N., A. B. Yongye, S. M. Tschampel, J. Gonzalez-Outeirino, C. R. Daniels, B. L. Foley and R. J. Woods. "Glycam06: A generalizable biomolecular force field. Carbohydrates." *Journal of Computational Chemistry* (2008): 29, 622-55. 10.1002/jcc.20820.
5. Zhang, J. and T. Lu. "Efficient evaluation of electrostatic potential with computerized optimized code." *Physical Chemistry Chemical Physics* (2021): 23, 20323-28. 10.1039/d1cp02805g.
6. D.A. Case, H. M. A., K. Belfon, I.Y. Ben-Shalom, J.T. Berryman, S.R. Brozell, D.S. Cerutti, T.E. Cheatham, III, G.A. Cisneros, V.W.D. Cruzeiro, T.A. Darden, R.E. Duke, G. Giambasu, M.K. Gilson, H. Gohlke, A.W. Goetz, R. Harris, S. Izadi, S.A. Izmailov, K. Kasavajhala, M.C. Kaymak, E. King, A. Kovalenko, T. Kurtzman, T.S. Lee, S. LeGrand, P. Li, C. Lin, J. Liu, T. Luchko, R. Luo, M. Machado, V. Man, M. Manathunga, K.M. Merz, Y. Miao, O. Mikhailovskii, G. Monard, H. Nguyen, K.A. O'Hearn, A. Onufriev, F. Pan, S. Pantano, R. Qi, A. Rahnamoun, D.R. Roe, A. Roitberg, C. Sagui, S. Schott-Verdugo, A. Shajan, J. Shen, C.L. Simmerling, N.R. Skrynnikov, J. Smith, J. Swails, R.C. Walker, J. Wang, J. Wang, H. Wei, R.M. Wolf, X. Wu, Y. Xiong, Y. Xue, D.M. York, S. Zhao, and P.A. Kollman. "Amber 2022." *University of California, San Francisco* (2022):

7. Maier, J. A., C. Martinez, K. Kasavajhala, L. Wickstrom, K. E. Hauser and C. Simmerling. "Ff14sb: Improving the accuracy of protein side chain and backbone parameters from ff99sb." *Journal of Chemical Theory and Computation* (2015): 11, 3696-713. 10.1021/acs.jctc.5b00255.

8. Wang, J. M., R. M. Wolf, J. W. Caldwell, P. A. Kollman and D. A. Case. "Development and testing of a general amber force field." *Journal of Computational Chemistry* (2004): 25, 1157-74. DOI 10.1002/jcc.20035.

9. Jorgensen, W. L., J. Chandrasekhar, J. D. Madura, R. W. Impey and M. L. Klein. "Comparison of simple potential functions for simulating liquid water." *Journal of Chemical Physics* (1983): 79, 926-35. Doi 10.1063/1.445869.

10. Andersen, H. C. "Molecular-dynamics simulations at constant pressure and-or temperature." *Journal of Chemical Physics* (1980): 72, 2384-93. Doi 10.1063/1.439486.

11. Darden, T., D. York and L. Pedersen. "Particle mesh ewald - an n.Log(n) method for ewald sums in large systems." *Journal of Chemical Physics* (1993): 98, 10089-92. Doi 10.1063/1.464397.

12. Miyamoto, S. and P. A. Kollman. "Settle - an analytical version of the shake and rattle algorithm for rigid water models." *Journal of Computational Chemistry* (1992): 13, 952-62. DOI 10.1002/jcc.540130805.

13. Roe, D. R. and T. E. Cheatham. "Ptraj and cpptraj: Software for processing and analysis of molecular dynamics trajectory data." *Journal of Chemical Theory and Computation* (2013): 9, 3084-95. 10.1021/ct400341p.

14. Kabsch, W. and C. Sander. "Dictionary of protein secondary structure - pattern-recognition of hydrogen-bonded and geometrical features." *Biopolymers* (1983): 22, 2577-637. DOI 10.1002/bip.360221211.
